# A systematic review of personality-related factors associated with autonomy-connectedness in functional neurological disorder

**DOI:** 10.3389/fpsyt.2026.1788675

**Published:** 2026-08-13

**Authors:** Luuk Stroink, Klaas Huijbregts, Andrej Rakic, Ralph de Vries, Marleen Rijkeboer, Marrie H. J. Bekker

**Affiliations:** 1 Mental Health Care Organization GGNet, Scelta, Department of Complex Trauma, Warnsveld, Netherlands; 2 Department of Clinical Psychology, Vrije Universiteit Amsterdam, Amsterdam, Netherlands; 3 Rehabilitation Centre Revalis, ‘s-Hertogenbosch, Netherlands; 4 Department of Clinical Psychological Science, Maastricht University, Maastricht, Netherlands

**Keywords:** alexithymia, autonomy-connectedness, conversion disorder, fear avoidance, functional neurological disorders, insecure attachment, interoceptive awareness, openness to experience

## Abstract

Preliminary evidence suggests that individuals with Functional Neurological Disorder (FND) may exhibit reduced autonomy-connectedness, a psychological construct referring to the capacity to remain connected with one’s own needs and emotions while maintaining meaningful relationships with others. Autonomy-connectedness consists of three interrelated dimensions: self-awareness, sensitivity to others, and the capacity to manage new situations. Although autonomy-connectedness itself has not yet been directly investigated in FND populations, several psychological characteristics associated with FND appear conceptually related to these dimensions, including insecure attachment, suggestibility, alexithymia, altered interoceptive awareness, disturbed sense of agency, and harm avoidance or low openness to experience. This systematic review examined empirical studies investigating psychological characteristics conceptually related to autonomy-connectedness in individuals with FND. Specifically, we reviewed studies on insecure attachment, suggestibility, alexithymia, interoception, sense of agency, and harm avoidance/openness to experience. Across studies, the most consistent associations were found for alexithymia and interoceptive disturbances, whereas findings regarding attachment, sense of agency, and harm avoidance/openness to experience were more heterogeneous. Evidence regarding suggestibility was limited. Overall, the findings provide indirect and hypothesis-generating support for the potential relevance of autonomy-connectedness as a conceptual framework for understanding vulnerability-related characteristics in FND. However, conclusions should be interpreted cautiously given the methodological heterogeneity across studies and the absence of direct assessments of autonomy-connectedness in FND populations.

**Systematic review registration:**

https://www.crd.york.ac.uk/PROSPERO/, identifier CRD42023232760.

## 1 Introduction

The first descriptions of personality-related characteristics in patients with hysteria and conversion disorder, now referred to as Functional Neurological Disorder (FND), emerged in the late nineteenth century. Since then, vulnerability-related characteristics associated with FND have been described from multiple theoretical perspectives, contributing to ongoing ambiguity regarding their clinical and theoretical significance (1, 2). Nevertheless, there is increasing recognition that such characteristics may play an important, yet underacknowledged, role in the development and maintenance of FND (3, 4). For example, attachment insecurity, emotional processing difficulties, and altered bodily self-awareness have all been implicated in FND and may exert transdiagnostic influences on symptom expression and illness course (5). At the same time, symptom-based classification approaches have not yet resulted in sufficient diagnostic agreement or consistently effective treatment strategies in FND (6, 7). A better understanding of vulnerability-related psychological characteristics may therefore contribute to more individualized and theoretically informed approaches to assessment and treatment (8).

**Figure 1:**
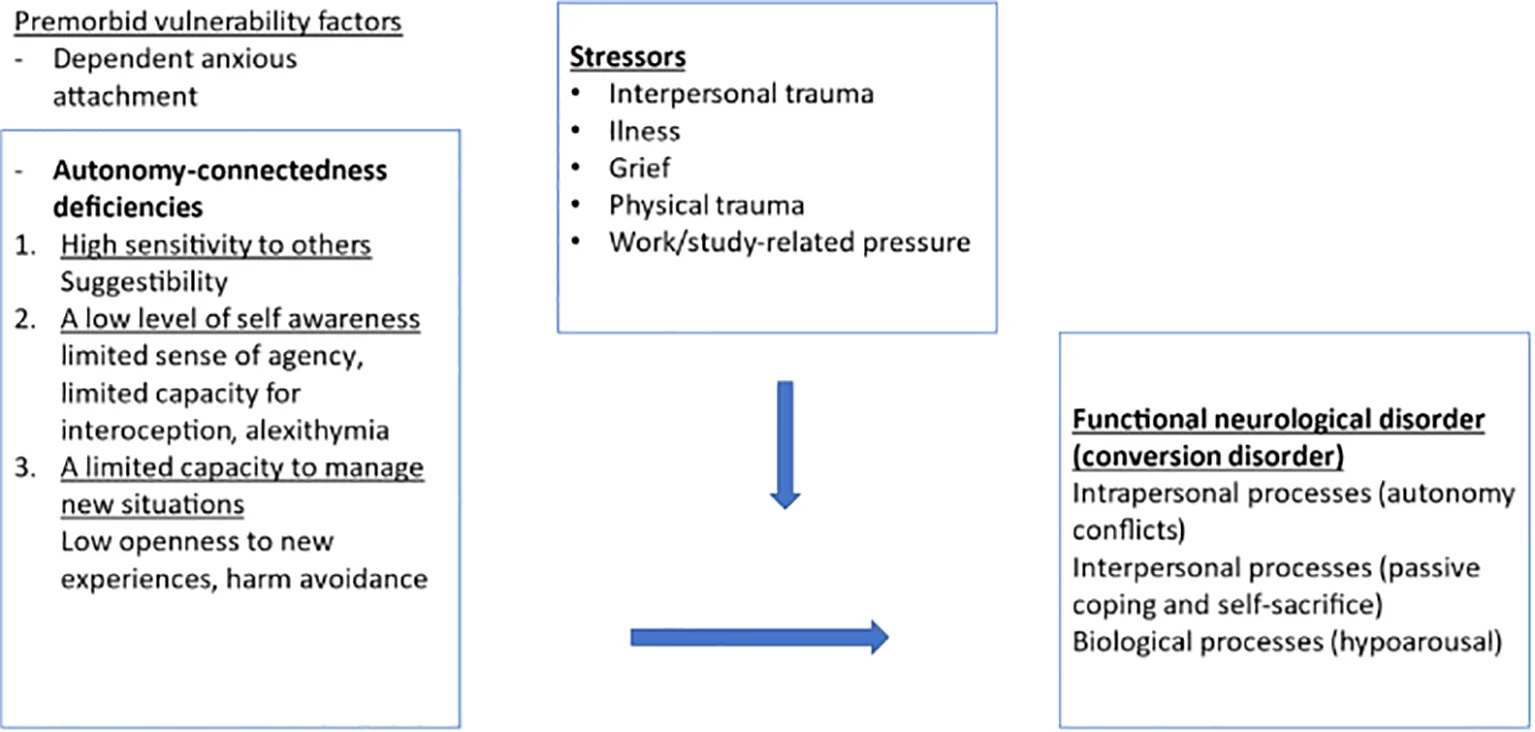
Hypothetical working model for functional neurological disorders (8, 10).

In the present review, we use the term personality-related factors to refer to relatively stable psychological characteristics that may reflect enduring patterns in emotional awareness, interpersonal functioning, embodied self-experience, and coping with novelty or uncertainty. These include insecure attachment, suggestibility, alexithymia, altered interoceptive awareness, disturbed sense of agency, harm avoidance, and low openness to experience. Although these constructs originate from different theoretical traditions, they may also be understood as conceptually related manifestations of a broader psychological framework: autonomy-connectedness (8, 9). **Figure 1** presents a hypothetical working model in which these characteristics are conceptually organized within the framework of autonomy-connectedness in relation to vulnerability for FND (8, 10).

Autonomy-connectedness refers to the capacity to remain connected with one’s own needs, wishes, and emotions while maintaining meaningful relationships with others (9). The construct consists of three interrelated dimensions: self-awareness, sensitivity to others, and the capacity to manage new situations (9, 11). Self-awareness refers to the ability to recognize and express one’s own wishes, needs, and emotions in interpersonal contexts. Sensitivity to others concerns awareness of and responsiveness to the wishes, needs, and emotions of others. The capacity to manage new situations refers to the extent to which individuals feel comfortable and capable in unfamiliar, unpredictable, or challenging situations. Conceptually, autonomy-connectedness is related to attachment theory (12), particularly the developmental process through which individuals learn to balance self-development and interpersonal connectedness within sufficiently safe relational environments.

Several psychological characteristics previously associated with FND appear conceptually related to these dimensions of autonomy-connectedness. For example, individuals with FND have been found to report higher levels of insecure attachment than healthy controls (13–15). Elevated suggestibility, although investigated in a limited number of studies, may reflect increased responsiveness to external cues and interpersonal influence, conceptually related to heightened sensitivity to others (16, 17). Furthermore, studies consistently report increased levels of alexithymia in individuals with FND (15, 18, 19), as well as reduced interoceptive awareness and disturbances in sense of agency (20–22). These findings may relate to difficulties in emotional awareness and embodied self-experience, conceptually linked to reduced self-awareness. In addition, reduced openness to experience and increased harm avoidance have been reported in several studies (23–25), potentially reflecting difficulties coping with uncertainty and novelty, conceptually related to a reduced capacity to manage new situations.

Importantly, the present review does not propose autonomy-connectedness as a replacement for existing explanatory models of FND, such as attachment-based, dissociative, emotion regulation, or predictive processing frameworks. Rather, autonomy-connectedness is explored here as a potentially integrative psychological framework through which vulnerability-related characteristics associated with FND may be conceptually organized. While several of the included constructs overlap conceptually and are not specific to FND, examining them together may help clarify recurring patterns in emotional, interpersonal, and embodied functioning relevant to this population. In this context, the predictive processing account proposed by Edwards et al. (26) may provide an important complementary framework, particularly regarding altered bodily predictions, agency, and symptom perception in FND.

Building on these observations and the hypothetical working model presented in **Figure 1**, the present review examines whether personality-related factors conceptually linked to autonomy-connectedness are associated with the presence of FND. Specifically, we review empirical studies investigating insecure attachment, suggestibility, alexithymia, interoception, sense of agency, harm avoidance, and openness to experience in individuals with FND. The first aim is to identify and synthesize empirical studies examining these constructs in relation to FND. The second aim is to evaluate the consistency and strength of the reported associations across studies. Importantly, the current review focuses specifically on vulnerability-related psychological characteristics. Although contextual factors such as trauma, physical illness, interpersonal stress, or loss are widely recognized as important in FND (27), their potential moderating or triggering role falls outside the scope of the present review.

Given that autonomy-connectedness has not yet been directly investigated in FND populations, the present review should be regarded as hypothesis-generating. We hypothesize that characteristics conceptually related to reduced autonomy-connectedness, including reduced self-awareness, heightened sensitivity to others, and difficulties managing novel or uncertain situations, are more prevalent in individuals with FND. By systematically synthesizing the existing literature, this review aims to evaluate the plausibility of autonomy-connectedness as a conceptual framework for understanding vulnerability-related characteristics in FND and to inform future empirical and clinical research.

## 2 Methods

This systematic review was conducted and reported in accordance with the Preferred Reporting Items for Systematic Reviews and Meta-Analyses (PRISMA) guidelines and checklist (28, www.prisma-statement.org). The review protocol was preregistered in PROSPERO on November 7, 2023. Study selection and screening were conducted using Rayyan (29, 30), and extracted study characteristics and outcomes were subsequently organized in Microsoft Excel. An updated literature search using the original search strategy was conducted in May 2026 prior to revision. The completed PRISMA checklist is provided in the **Supplementary Materials**.

### 2.1 PICOS framework

The review question was formulated using the PICOS framework. Participants included adults (≥18 years) diagnosed with Functional Neurological Disorder (FND) and/or Psychogenic Non-Epileptic Seizures (PNES), as defined according to the diagnostic criteria used in the original studies. The exposure of interest comprised psychological characteristics conceptually related to autonomy-connectedness, including insecure attachment, alexithymia, suggestibility, altered interoceptive awareness, disturbed sense of agency, harm avoidance, and low openness to experience. Comparators included healthy control groups and, where applicable, additional neurological or psychiatric comparison groups. Outcomes included group differences and reported associations between FND and the included psychological characteristics. Eligible study designs included observational studies, such as cross-sectional, case–control, and cohort studies.

### 2.2 Search strategy

To identify relevant studies, systematic searches were conducted in the bibliographic databases Ovid MEDLINE and APA PsycInfo (EBSCO) from inception to September 6, 2024, in collaboration with a medical information specialist. An updated search using the original search strategy was subsequently conducted in May 2026 to identify newly published studies. In addition, Google Scholar was consulted to screen for potentially relevant recent publications and grey literature. This update identified several additional potentially relevant records, primarily concerning alexithymia, interoception, attachment/personality characteristics, and sense of agency. No studies directly investigating autonomy-connectedness in FND populations were identified. Overall, the additional literature did not substantially alter the general pattern of findings or the overall interpretation of the review.

The search strategy combined terms related to Functional Neurological Disorder (FND) with terms related to psychological characteristics conceptually linked to autonomy-connectedness. FND-related search terms included, among others, “functional neurological disorder,” “conversion disorder,” “psychogenic non-epileptic seizures,” “functional movement disorder,” “FND,” “FNSD,” and related Medical Subject Headings (MeSH) terms. These were combined using Boolean operators (“AND”/”OR”) with terms related to insecure attachment, suggestibility, sensitivity to others, interoception, alexithymia, sense of agency, harm avoidance, openness to experience, and autonomy-connectedness itself. Both controlled vocabulary terms (e.g., MeSH terms) and free-text terms, including synonyms and related concepts, were used.

Duplicate records were removed by a medical information specialist using EndNote X21.0.1 (Clarivate™), following the Amsterdam Efficient Deduplication (AED)-method (31) and the Bramer-method (32). The complete search strategies for all databases are provided in **Supplementary Appendix B**.

### 2.3 Study selection

Titles and abstracts identified through the database searches were screened independently by the first author (LS). Studies were considered eligible if they investigated psychological characteristics conceptually related to autonomy-connectedness in adults (≥18 years) with Functional Neurological Disorder (FND), Psychogenic Non-Epileptic Seizures (PNES), or conversion disorder. Diagnostic classifications were accepted as defined in the original studies. In studies using older terminology such as hysteria, diagnostic descriptions were evaluated for consistency with DSM-5 criteria for FND/conversion disorder (33).

Studies were included if they examined direct associations between FND and at least one of the predefined psychological characteristics of interest: insecure attachment, suggestibility, alexithymia, interoception, sense of agency, harm avoidance, or openness to experience. To improve comparability across studies and reduce potential confounding, studies including healthy control groups were preferentially included. Additional neurological or psychiatric comparison groups were included where available.

Eligible study designs included observational studies, such as cross-sectional, longitudinal, cohort, and case–control studies. Experimental and quasi-experimental studies were also considered when relevant baseline associations with the constructs of interest were reported. Case reports and case series were excluded due to limited generalizability and statistical power. Studies were additionally excluded if they did not address the review question, did not investigate direct associations with the predefined psychological characteristics, or did not use validated assessment instruments.

**Figure 2:**
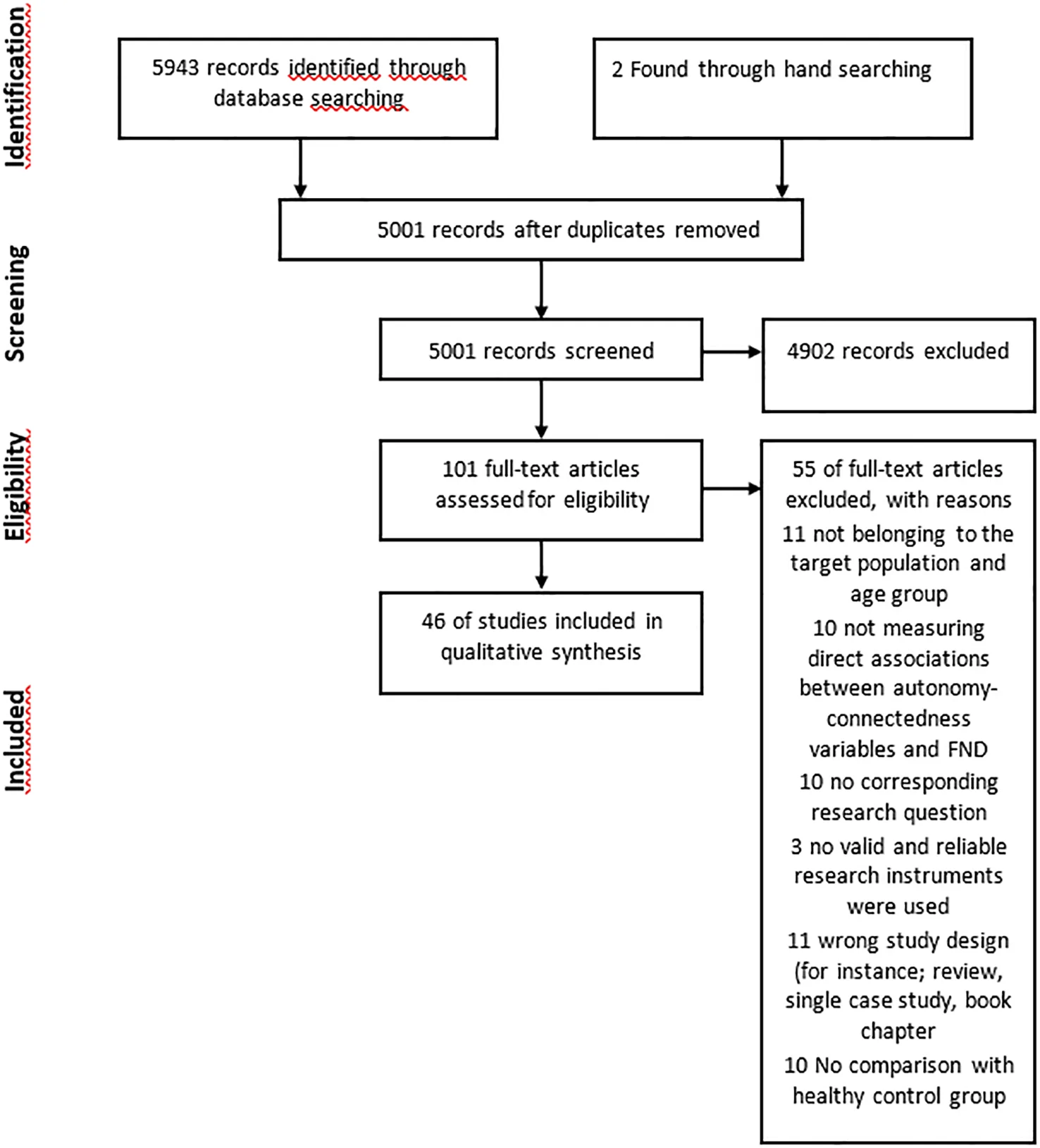
PRISMA flow chart of the study outcome selection.

Following title and abstract screening, all potentially eligible studies were retrieved in full text and assessed for final inclusion. The study selection process is presented in the PRISMA flowchart (**Figure 2**).

### 2.4 Risk of bias assessment

Most included studies were cross-sectional; therefore, the Appraisal Tool for Cross-Sectional Studies (AXIS; 34) was used to assess methodological quality and risk of bias. The AXIS tool evaluates several aspects of study quality, including statistical power, sampling procedures, measurement quality, consistency of statistical analyses, reporting transparency, and other potential sources of bias.

Quality assessment was conducted independently by two authors (LS and KH). To assess the reliability of the ratings, inter-rater reliability was calculated using both the Intraclass Correlation Coefficient (ICC) and Cohen’s kappa. Initial agreement between raters was low to fair (ICC = 0.21), while Cohen’s kappa indicated a fair level of agreement [κ = 0.31, 95% CI (0.02, 0.61)]. These findings suggest some variability in the interpretation of AXIS criteria across studies. To address this, discrepancies were subsequently discussed and resolved through consensus, with involvement of a third reviewer (AR) where necessary, in accordance with common practice in systematic reviews (35). Final risk of bias ratings was therefore based on consensus judgments.

The AXIS criteria covered the following aspects: clarity of aims and objectives, appropriateness of study design, justification of the sampling framework, rationale for sample size, measures addressing non-response and response bias, evaluation of outcome variables and risk factors, clarity of methods and statistical approach, presentation of results, justification of conclusions, acknowledgment of limitations, and reporting of ethical approval and conflicts of interest.

Each study was evaluated against these criteria to determine its methodological quality. Based on the overall AXIS assessment, studies were categorized as having low, moderate, or high risk of bias. These quality assessments were subsequently taken into account when interpreting and synthesizing the findings across studies.

### 2.5 Data extraction and synthesis

Data from the included studies were independently extracted by two authors (LS and KH) using a Microsoft Excel extraction sheet. Extracted study characteristics included first author, publication year, study design (e.g., cross-sectional or prospective observational study), study population, sample characteristics (e.g., sex, ethnicity, age), diagnostic criteria, and assessment instruments. The extracted variables included psychological characteristics conceptually related to autonomy-connectedness, such as suggestibility, alexithymia, interoception, sense of agency, openness to experience, and harm avoidance. In addition, findings relevant to the current review, including main conclusions and statistical parameters (e.g., test statistics, p-values, and handling of missing values or outliers), were documented.

Where possible, effect sizes (e.g., Cohen’s d and odds ratios) were extracted or calculated to facilitate comparison across studies and to evaluate the strength of reported associations. Specifically, effect sizes were examined for differences in autonomy-connectedness-related characteristics between individuals with FND and comparison groups, including healthy control groups where available. Effect sizes were interpreted according to Cohen’s guidelines: d = 0.2 as small, d = 0.5 as medium, and d = 0.8 as large; for correlations, r = 0.10 was considered small, r = 0.30 medium, and r = 0.50 large (36).

If studies did not report effect sizes directly, Cohen’s d was calculated by LS using reported t-statistics or, when unavailable, group means and standard deviations. Statistical analyses and effect size calculations were performed using SPSS.

Due to substantial heterogeneity in study designs, populations, and outcome measures, a meta-analysis was not conducted. Instead, a narrative synthesis approach was used to integrate findings across studies (37), allowing for a qualitative interpretation of patterns and methodological differences in the literature.

## 3 Results

This section presents the findings of the included studies, organized according to the psychological characteristics conceptually related to autonomy-connectedness: insecure attachment, suggestibility, interoception, alexithymia, sense of agency, harm avoidance, and openness to experience. For each construct, we describe the characteristics of the included studies, study populations, methodological quality, and main findings. Particular attention is given to the direction, strength, and consistency of reported associations with FND, as well as methodological limitations and potential sources of bias across studies.

### 3.1 Attachment (e.g., attachment styles)

#### 3.1.1 Quality and bias assessment

Six studies investigated attachment-related characteristics (e.g., attachment styles) in individuals with Functional Neurological Disorder (FND). Study designs included one case–control study, one cross-sectional study, two prospective cohort studies, and two retrospective studies. The studies were conducted in the United Kingdom (n = 2), Italy (n = 2), Germany (n = 1), and the Czech Republic (n = 1). Across studies, approximately 75% of participants were female. No major inconsistencies were identified regarding demographic characteristics, such as ethnicity, educational background, employment status, or clinical presentation of FND. An overview of the included studies is provided in **Supplementary Appendix A**.

Quality appraisal revealed several recurring methodological limitations. Most notably, all studies showed an increased risk of non-response bias. In addition, four of the six studies did not provide adequate justification for sample size, nor did they consistently report psychometric properties such as internal consistency measures (e.g., Cronbach’s alpha). Incomplete reporting of study outcomes was also observed in several studies.

At the same time, most studies demonstrated relatively low risk of bias in key methodological domains, including clarity of study aims, appropriateness of the target population, participant selection procedures, transparency of statistical analyses, and acknowledgment of study limitations. Overall, the methodological quality of the included attachment studies was considered moderate, although variability in reporting quality and potential response bias should be taken into account when interpreting the findings.

#### 3.1.2 Results: data extraction and synthesis

All six studies examined associations between insecure attachment and Functional Neurological Disorder (FND), typically by comparing individuals with FND or Psychogenic Non-Epileptic Seizures (PNES) to healthy control groups. Across studies, insecure attachment patterns were consistently reported more frequently in individuals with FND, although the specific attachment styles and assessment methods varied considerably.

Cuoco et al. (13), using the Experiences in Close Relationships-Revised (ECR-R; 38), found significantly higher levels of avoidant attachment in individuals with FND compared to both neurological and healthy controls. Avoidant attachment independently predicted depressive symptoms and alexithymia and contributed significantly to FND group classification in logistic regression analyses (β = 0.88, p = .008, R^2^ = .51).

Gerhardt et al. (14), using the Adult Attachment Projective Picture System (AAP; 39), reported a significant association between disorganized attachment and FND (OR = 2.59). Similarly, Kramska et al. (40), using the Experiences in Close Relationships-Relationship Structures questionnaire (ECR-RS; 41), found a moderate association between preoccupied attachment and PNES (Cohen’s d = 0.41).

Levita et al. (42), using the Relationship Scales Questionnaire (RSQ), observed significantly higher levels of anxious and avoidant attachment in individuals with FND compared to controls. However, relationship insecurity showed only a modest association with FND group status in logistic regression analyses, which did not remain significant after correction for multiple comparisons.

Williams et al. (15) found that fearful attachment was associated with alexithymia, depression, dissociation, and childhood trauma in patients with motor FND. In multivariate analyses, childhood abuse, alexithymia, and depressive symptoms independently predicted fearful attachment. Jalilianhasanpour et al. (43), also using the RSQ, reported a significant positive association between insecure attachment and FND (β = 0.46).

Overall, the included studies suggest that insecure attachment may be more prevalent in individuals with FND compared to healthy controls. However, the findings were heterogeneous with respect to attachment subtypes, assessment methods, and associated psychological characteristics. While avoidant, anxious, fearful, preoccupied, and disorganized attachment patterns were all implicated across studies, no single attachment profile appeared uniquely characteristic of FND. These findings may indicate that different forms of attachment insecurity are associated with vulnerability-related psychological processes relevant to FND, although the specificity and underlying mechanisms of these associations remain unclear.

### 3.2 Suggestibility

#### 3.2.1 Quality and bias assessment

Two studies investigated suggestibility in individuals with Functional Neurological Disorder (FND), including one case–control study and one cross-sectional study. The studies were conducted in the Netherlands and the United Kingdom. Limited information was provided regarding ethnicity and racial background. Across studies, approximately 75% of participants were female. No major outliers were identified regarding age or educational level. An overview of the included studies is provided in **Supplementary Appendix A**.

Quality assessment indicated several methodological limitations. Both studies showed an increased risk of non-response bias and were characterized by relatively small sample sizes. In addition, reporting of descriptive statistics and methodological details was limited in one study. The studies also differed substantially in their operationalization and assessment of suggestibility, complicating direct comparison of findings. Overall, the methodological quality of the included suggestibility studies was considered moderate to low.

#### 3.2.2 Results: data extraction and synthesis

Two studies examined associations between suggestibility and FND, both comparing individuals with FND to healthy control groups. However, the studies differed substantially in how suggestibility was conceptualized and measured.

Huys et al. (44) investigated suggestibility using a classical placebo paradigm and found no significant differences in placebo responsiveness between individuals with FND and healthy controls. Although the findings were non-significant, a small effect size was reported (η^2^ = 0.038).

In contrast, Roelofs et al. (16) assessed hypnotic suggestibility and found higher levels of suggestibility in individuals with FND compared to healthy controls, with a medium effect size (d = 0.6).

Taken together, the available evidence regarding suggestibility in FND remains limited and heterogeneous. While one study reported increased hypnotic suggestibility in individuals with FND, the other did not find significant differences in placebo responsiveness. These inconsistencies may partly reflect differences in the operationalization and measurement of suggestibility across studies. Given the small number of available studies and the methodological variability, conclusions regarding the role of suggestibility in FND should be interpreted cautiously.

### 3.3 Alexithymia

#### 3.3.1 Quality and bias assessment

All 20 included studies investigated associations between alexithymia and Functional Neurological Disorder (FND), typically by comparing individuals with FND to healthy control groups. Study designs included cross-sectional (n = 12), case–control (n = 7), and exploratory designs (n = 1). Most studies were conducted in English-speaking countries (n = 9), with additional studies from Germany (n = 5), the Czech Republic (n = 2), and single studies from the Netherlands, France, Israel, and Spain. Across studies, the weighted mean proportion of female participants was 75.31%, although one study included only male participants.

Quality assessment indicated that most studies demonstrated moderate methodological quality. Non-response bias was identified in 18 of the 20 studies, and many studies lacked adequate justification of sample size. Approximately half of the studies did not report internal consistency measures for the assessment instruments used. At the same time, a major strength across studies was the consistent use of validated and widely used alexithymia measures, particularly the Toronto Alexithymia Scale (TAS). Overall, methodological quality was considered moderate, although potential response bias and variability in reporting quality should be taken into account when interpreting the findings.

#### 3.3.2 Results: data extraction and synthesis

All included studies reported positive associations between alexithymia and FND. Across studies, effect sizes for total alexithymia scores ranged from moderate to very large (Cohen’s d = 0.62–1.94), suggesting consistently elevated alexithymia levels in individuals with FND compared to healthy controls.

Six studies additionally examined alexithymia subdomains, including difficulty identifying feelings, difficulty describing feelings, and externally oriented thinking. Across these studies, the most consistent findings were observed for difficulty identifying feelings and difficulty describing feelings. Individuals with FND generally reported significantly greater difficulty recognizing and verbalizing emotions than healthy controls, with effect sizes ranging from moderate to large across most studies. For example, Gulpek et al. (19), Demartini et al. (45), Herrero et al. (46), and Cole et al. (47) all reported significant group differences across these domains.

In contrast, findings regarding externally oriented thinking were less consistent. While some studies reported small to moderate group differences, others found no significant differences between individuals with FND and healthy controls. This suggests that externally oriented thinking may be less strongly or less consistently associated with FND than the emotional awareness components of alexithymia.

**Figure 3:**
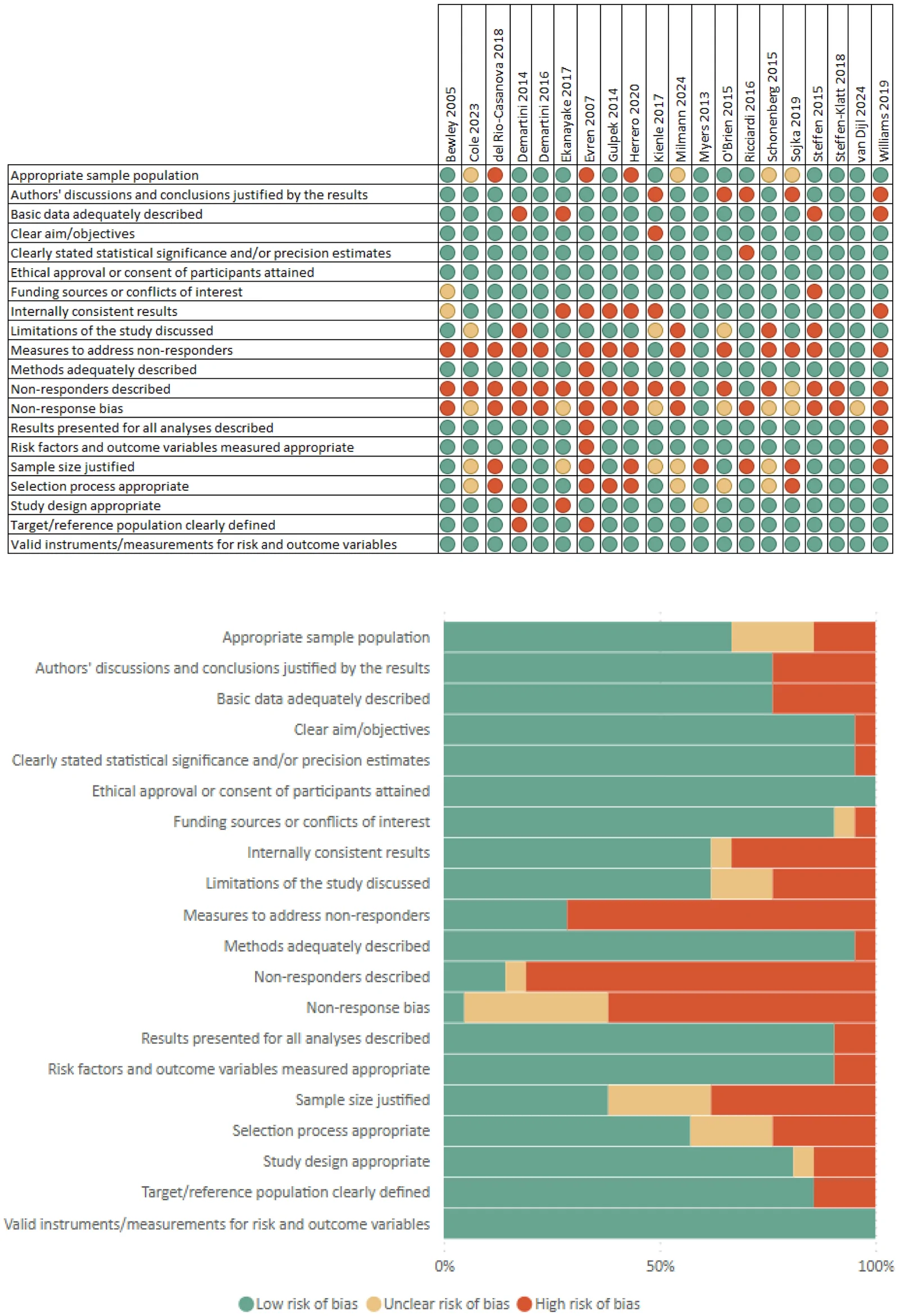
Risk of bias summary for studies on alexithymia.

Taken together, the available evidence suggests a relatively robust association between alexithymia and FND, particularly regarding difficulties identifying and describing feelings. However, the findings should be interpreted cautiously given the methodological heterogeneity across studies, variability in effect sizes, and the limited use of correction procedures for multiple testing. The overall risk of bias assessment for the included alexithymia studies is presented in **Figure 3**.

### 3.4 Interoception

#### 3.4.1 Quality and bias assessment

Seven studies investigated interoception in individuals with Functional Neurological Disorder (FND), including two case–control studies and five cross-sectional studies. The studies were conducted across several countries, including the Czech Republic, the United Arab Emirates, and multiple English-speaking countries. Across studies, female participants predominated, ranging from 62% to 79% of samples, with an average female representation of approximately 70%. No major demographic outliers were identified regarding age, ethnicity, or educational level. An overview of the included studies is provided in **Supplementary Appendix A**.

Quality assessment revealed several recurring methodological limitations. Non-response bias was identified across all studies, largely due to limited reporting regarding non-responders. In addition, most studies used relatively small sample sizes and did not provide formal power analyses or sample size justifications. Nevertheless, all studies used validated and reproducible instruments to assess interoceptive functioning, and study objectives and methodologies were generally described clearly. Overall, the methodological quality of the included studies was considered moderate, although the identified risks of bias should be taken into account when interpreting the findings.

#### 3.4.2 Results: data extraction and synthesis

Seven studies examined associations between interoception and FND, assessing both interoceptive accuracy and interoceptive sensibility using a variety of experimental and self-report measures.

Interoceptive accuracy refers to the objective ability to detect internal bodily signals, such as heartbeat perception. Several studies reported reduced interoceptive accuracy in individuals with FND compared to healthy controls. For example, Koreki et al. (22), using the Heartbeat Tracking Task (HTT; 48), found significantly lower interoceptive accuracy scores in individuals with FND (d = 1.12, p <.001). Similarly, Pick et al. (1) reported a moderate effect size (d = 0.38, p = .021). Sojka et al. (2020) also reported a large effect size, although group differences did not reach statistical significance, likely due to the small sample size.

Interoceptive sensibility refers to the subjective perception and interpretation of bodily sensations. Several studies found altered self-reported interoceptive functioning in individuals with FND. Koreki et al. (22), using the Body Perception Questionnaire (BPQ), found significantly higher bodily awareness scores in individuals with FND compared to controls (d = 1.61, p <.001). Studies using the Multidimensional Assessment of Interoceptive Awareness (MAIA-2) similarly reported impairments on subdomains such as “not distracting” and “trusting” (1, 49), suggesting difficulties in adaptive engagement with bodily sensations. Demartini et al. (45) additionally reported significantly reduced interoceptive functioning in individuals with FND compared to healthy controls, with a large effect size (d = 0.86).

Two studies also examined Interoceptive Trait Prediction Error (ITPE), reflecting discrepancies between subjective and objective interoceptive functioning. Koreki et al. (22) reported significantly elevated ITPE scores in individuals with FND (d = 1.61, p <.001), suggesting greater mismatch between perceived and objectively measured bodily awareness.

Overall, the available evidence suggests relatively consistent associations between altered interoceptive functioning and FND. Across studies, individuals with FND generally demonstrated reduced interoceptive accuracy alongside disturbances in subjective bodily awareness and interpretation. However, findings should be interpreted cautiously given the methodological heterogeneity across studies, small sample sizes, and variability in the operationalization of interoception.

### 3.5 Sense of agency

#### 3.5.1 Quality and bias assessment

Seven studies investigated sense of agency (SA) in individuals with Functional Neurological Disorder (FND), primarily using cross-sectional designs. The studies were conducted in Italy and the United Kingdom. Across studies, female participants predominated, ranging from approximately 70% to 90% of the samples. No major demographic outliers were identified regarding age, ethnicity, or educational level. An overview of the included studies is provided in **Supplementary Appendix A**.

Quality assessment revealed several methodological limitations. Most studies showed an increased risk of non-response bias and provided limited information regarding non-responders. In addition, descriptions of study populations and basic demographic characteristics were often limited. Sample sizes were generally small, and methodological approaches varied considerably across studies. Nevertheless, the included studies employed a range of established experimental paradigms to assess sense of agency and related sensorimotor processes. Overall, methodological quality was considered moderate, although variability in operationalization and study design should be taken into account when interpreting the findings.

#### 3.5.2 Results: data extraction and synthesis

Seven studies examined sense of agency (SA) in individuals with FND using a range of experimental paradigms assessing action–outcome relationships, sensory attenuation, body ownership, movement perception, and proprioceptive processing. Across studies, altered sense of agency was generally observed in individuals with FND compared to healthy controls.

Several studies investigated action–outcome integration and sensory attenuation. For example, Kranick et al. (50), using an action–effect binding paradigm, found that individuals with FND perceived weaker associations between voluntary actions and their consequences compared to healthy controls (d = 0.81, p = .005). Similarly, Macerollo et al. (51) and Pareés et al. (52) reported abnormalities in sensory attenuation paradigms, suggesting altered processing of self-generated sensory input in individuals with FND.

Other studies focused on body ownership, movement perception, and proprioceptive integration. Marotta et al. (53), using the rubber hand illusion paradigm, found stronger suppression of agency during passive movement conditions in individuals with FND. Nahab et al. (21), combining functional MRI with a virtual reality movement task, reported persistent experiences of agency over externally controlled movements, with a large effect size (Cramer’s V = 0.827). Tinazzi et al. (54) additionally reported impairments in proprioceptive processing and sensory integration using a tendon vibration paradigm.

Overall, the available evidence suggests that altered sense of agency may be associated with FND across a range of experimental paradigms. However, substantial methodological heterogeneity was present regarding the operationalization and assessment of sense of agency. In addition, most studies used relatively small sample sizes and cross-sectional designs. Consequently, although findings were generally consistent in suggesting altered agency-related processing in FND, conclusions regarding the specificity and underlying mechanisms of these alterations should be interpreted cautiously.

### 3.6 Harm avoidance and openness to experience

#### 3.6.1 Quality and bias assessment

Six studies investigated harm avoidance and openness to experience in individuals with Functional Neurological Disorder (FND). All studies used cross-sectional designs, including one retrospective case–control study. Most studies were conducted in English-speaking countries, including the United States and Australia, with additional studies from the Netherlands and Turkey. No major demographic outliers were identified regarding age, ethnicity, educational level, or clinical characteristics. Across studies, female participants predominated, ranging from 62% to 80% of the samples. An overview of the included studies is provided in **Supplementary Appendix A**.

Quality assessment revealed several methodological limitations. All studies showed an increased risk of non-response bias and provided limited information regarding non-responders. In addition, evidence regarding internal consistency was often insufficiently reported. Nevertheless, all studies used validated instruments to assess personality-related characteristics and outcome variables. Overall, the methodological quality of the included studies was considered moderate, although the identified risks of bias should be taken into account when interpreting the findings.

#### 3.6.2 Results: data extraction and synthesis

Six studies examined harm avoidance and openness to experience in individuals with FND. The most commonly used instruments were the Temperament and Character Inventory (TCI; 55), the Tridimensional Personality Questionnaire (TPQ; 56), and the NEO personality inventories (57).

Several studies reported elevated harm avoidance in individuals with FND compared to healthy controls. Erten et al. (24), using the TCI, found significantly higher harm avoidance scores in individuals with FND, with a large effect size (d = 1.28). Similarly, Morris et al. (25), using an avoidance learning task combined with functional MRI, reported elevated harm avoidance with a large effect size (Hedges’ g = 0.88). Sarisoy et al. (23) also found higher harm avoidance and lower novelty seeking in individuals with FND, although effect sizes were smaller (d = 0.30–0.33). Willinger et al. (58), using the TPQ, similarly reported moderate differences in harm avoidance (d = 0.49), while differences in novelty seeking were relatively small (d = 0.19).

In contrast, Ekanayake et al. (59) and Leong et al. (60), both using NEO-based personality measures, did not find significant differences between individuals with FND and healthy controls regarding openness to experience or related personality dimensions.

Overall, findings regarding harm avoidance and openness to experience were mixed. Several studies, particularly those using the TCI or TPQ, reported elevated harm avoidance and reduced novelty seeking in individuals with FND. However, studies using broader five-factor personality models did not consistently replicate these findings. These inconsistencies may partly reflect differences in the conceptualization and operationalization of personality traits across assessment instruments. Consequently, the evidence for altered harm avoidance and openness to experience in FND should be interpreted cautiously.

## 4 Discussion

This systematic review examined the empirical associations between Functional Neurological Disorder (FND) and psychological characteristics conceptually related to autonomy-connectedness. Specifically, we reviewed studies investigating insecure attachment, suggestibility, alexithymia, interoception, sense of agency, harm avoidance, and openness to experience. These constructs can be conceptually related to the three dimensions of autonomy-connectedness: self-awareness, sensitivity to others, and the capacity to manage new situations. Taken together, the findings provide preliminary support for the hypothesis that reduced autonomy-connectedness may represent a relevant vulnerability-related psychological dimension in individuals with FND, rather than evidence for a direct causal mechanism. At the same time, the consistency and strength of associations varied considerably across constructs (61–63).

The most consistent findings emerged for alexithymia and interoception. Alexithymia was significantly elevated in the majority of studies, suggesting that difficulties identifying and verbalizing emotions may represent an important vulnerability-related characteristic in FND (15, 18, 19). Within the autonomy-connectedness framework, emotional awareness is conceptually related to the broader dimension of self-awareness, which includes not only awareness of internal states, but also the capacity to recognize, express, and act upon one’s needs and emotions in interaction with others (64). This broader relational dimension may be particularly relevant in FND, where interpersonal and emotional processes are often intertwined with symptom expression. Similarly, studies on interoception indicated disturbances in both interoceptive accuracy and interoceptive sensibility, with several studies reporting impairments in perceiving, interpreting, or trusting bodily signals (1, 20, 22, 49). Together, these findings may point toward disturbances in embodied self-awareness in individuals with FND.

Other constructs yielded more heterogeneous findings. Studies examining sense of agency generally suggested altered agency-related processing in individuals with FND, including abnormalities in action–outcome integration, sensory attenuation, body ownership, and proprioceptive processing (21, 50–54). However, substantial methodological heterogeneity across paradigms makes it difficult to draw firm conclusions regarding the specificity or underlying mechanisms of these findings. Findings regarding attachment insecurity were similarly variable. Although several studies reported elevated insecure attachment patterns, including avoidant, fearful, preoccupied, and disorganized attachment (13–15, 40), no single attachment profile appeared uniquely characteristic of FND. Findings for suggestibility, harm avoidance, and openness to experience were mixed or limited. Roelofs et al. (16) reported increased hypnotic suggestibility in individuals with FND, whereas Huys et al. (44) found no significant differences using a placebo responsiveness paradigm. Similarly, studies investigating harm avoidance and novelty seeking reported inconsistent findings, with some studies demonstrating elevated harm avoidance (23–25), while others using broader five-factor personality models found no significant differences (59, 60). These inconsistencies may partly reflect methodological and conceptual differences in how these constructs are operationalized and assessed.

Several methodological limitations complicate interpretation of the reviewed literature. Most included studies used cross-sectional designs, precluding conclusions regarding temporal or causal relationships. Consequently, it remains unclear whether the identified characteristics reflect vulnerability-related factors, consequences of living with FND, or reciprocal processes that evolve over time. In addition, many studies relied heavily on self-report instruments, which may be limited in capturing pre-reflective bodily processes and non-verbal emotional experiences relevant to FND symptomatology. Although some studies employed experimental paradigms, methodological heterogeneity remained substantial across domains such as interoception, sense of agency, and suggestibility. Psychiatric comorbidities were also not consistently controlled for, making it difficult to determine whether the observed associations are specific to FND or reflect broader transdiagnostic vulnerabilities. Furthermore, many studies were characterized by relatively small sample sizes, high non-response bias, and variability in methodological quality. Even in domains where findings appeared relatively consistent, such as alexithymia and interoception, these methodological limitations warrant cautious interpretation.

Conceptually, several of the reviewed constructs may not be best understood as entirely stable personality traits, but rather as context-sensitive relational and regulatory patterns. For this reason, the present review adopted the broader term “personality-related factors,” reflecting a continuum ranging from relatively enduring dispositions to more dynamic psychological vulnerabilities. Within the context of FND, these characteristics appear relevant to both intrapersonal functioning (e.g., emotional awareness, bodily perception, and agency) and interpersonal functioning (e.g., attachment and sensitivity to others).

From a clinical perspective, the findings may have implications for transdiagnostic treatment approaches targeting emotional awareness, bodily awareness, self-regulation, and relational functioning. In particular, the relatively consistent findings regarding alexithymia and interoception suggest that interventions aimed at improving emotional and embodied self-awareness may be relevant for individuals with FND. Examples may include mindfulness-based interventions, affect labeling, body-oriented psychotherapies, and approaches targeting interoceptive awareness. In addition, interventions addressing attachment-related difficulties and relational safety may also be clinically relevant in subgroups of individuals with elevated attachment insecurity. Within this context, Autonomy-Enhancing Treatment (AET), which explicitly focuses on strengthening self-awareness, relational functioning, and flexibility in managing new situations, may represent a theoretically congruent treatment framework. However, its relevance for FND currently remains theoretical and hypothesis-generating, as direct intervention studies in FND populations are still lacking.

Future research should prioritize longitudinal, experimental, and multi-method designs to clarify the temporal and causal relationships between psychological vulnerabilities and FND. Greater methodological consistency in the operationalization of constructs such as suggestibility, interoception, and sense of agency is also needed. In addition, future studies should include larger and more diverse samples, employ clinical comparison groups, and more consistently account for psychiatric comorbidity. The use of complementary assessment approaches, including behavioral, physiological, and experimental paradigms alongside self-report measures, may further improve understanding of the embodied and relational processes involved in FND. Clarifying the potential role of autonomy-connectedness within these processes may contribute to more refined conceptual models and more personalized treatment approaches for individuals with FND (65).

## 5 Conclusion

This review is the first to systematically synthesize empirical studies examining psychological characteristics conceptually related to autonomy-connectedness in individuals with Functional Neurological Disorder. Overall, the findings suggest that characteristics such as alexithymia, altered interoceptive functioning, insecure attachment, and disturbances in sense of agency are associated with FND, providing preliminary support for the hypothesis that reduced autonomy-connectedness may represent a relevant psychological dimension in FND. In particular, the relatively consistent associations observed for alexithymia and interoception suggest that emotional and embodied self-awareness may represent important vulnerability-related domains in individuals with FND.

At the same time, the methodological quality of the included studies was variable and frequently limited by small sample sizes, cross-sectional designs, high risk of non-response bias, and inconsistent control for psychiatric comorbidity. Furthermore, most studies compared individuals with FND primarily to healthy control groups, limiting conclusions regarding the specificity of findings to FND relative to other psychiatric or neurological conditions. Consequently, the current findings should be interpreted cautiously and regarded as hypothesis-generating rather than confirmatory.

Future research should employ longitudinal, experimental, and multi-method approaches to further investigate the role of autonomy-connectedness-related characteristics in FND. Such work may contribute to more refined vulnerability models and support the development of more personalized and integrative approaches to assessment and treatment in FND.

## Data Availability

The raw data supporting the conclusions of this article will be made available by the authors, without undue reservation.
