# Supplementary material for: A systematic review of personality-related factors associated with autonomy-connectedness in functional neurological disorder
Source: Front Psychiatry. 2026 Aug 13;17:1788675. doi: 10.3389/fpsyt.2026.1788675 (PMC13519597; doi:10.3389/fpsyt.2026.1788675)
Supplement: Supplementary file 1 [file SupplementaryFile1.docx]

**Appendix A.**

**Attachment**

*Figure 4. Risk of bias summary attachment*

**Suggestibility**

*Figure 5. Risk of bias summary suggestibility*

**Interoception**

*Figure 6. Risk of bias summary interoception*

**Sense of agency**

*Figure 7. Riks of bias summary sense of agency*

**Harm avoidance/openness to new experiences**

*Figure 8. Risk of bias summary harm avoidance/openness to new experiences*

**Appendix B**

**Ovid Medline Search Results – 06 Sep 2024** 

| **Search** | **Query** | **Results** |
| --- | --- | --- |
| **#3** | **1 and 2** | **3,754** |
| **#2** | **"Personality"/ or exp "Personal Autonomy"/ or "Internal-External Control"/ or "Affective Symptoms"/ or "Interoception"/ or "Harm Reduction"/ or ("personality style*" or "personality trait*" or "attachment" or "suggestibility" or ("sensitivity" adj3 "other*") or "interoceptive awareness" or "interoception*" or "alexithymia" or ("sens*" adj3 "agenc*") or ("open*" adj3 "experienc*") or ("harm*" adj3 ("avoid*" OR "reduct*")) or "agreeable*" or "autonom* connect*").ab,ti,kf.** | **259,279** |
| **#1** | **"Somatoform Disorders"/ or "Psychophysiologic Disorders"/ or exp "Conversion Disorder"/ or exp "Factitious Disorders"/ or exp "Dissociative Disorders"/ or (exp "Vision Disorders"/ and "functional".ab,ti,kw.) or ("Motor Disorders"/ and "functional".ab,ti,kw.) or exp "Histrionic Personality Disorder"/ or "Catalepsy"/ or ("conversion disorder*" or "somatoform dis*" or "functional tic*" or "functional paralys*" or "functional vis*" or "hysterical paralys*" or "hysterical vision disturbance*" or "hysterical blindness" or "hysteria*" or "histrionic" or "pseudodementia" or "pseudo-dementia" or "catalep*" or "psychogenic nonepileptic seizur*" or "psychogenic non-epileptic seizur*" or "PNES" or ("functional neurological" adj3 "disorder*") or "FND" or "FNSD" or "function* motor disorder*" or "FMD").ab,ti,kf.** | **66,368** |

**APA PsycInfo (Ebsco) Search Results – 06 Sep 2024** 

| **Search** | **Query** | **Results** |
| --- | --- | --- |
| **S3** | **S1 AND S2** | **2,189** |
| **S2** | **DE "Personality Traits" OR DE "Independence (Personality)" OR DE "Internal External Locus of Control" OR DE "Alexithymia" OR DE "Suggestibility" OR DE "Interoception" OR DE "Harm Reduction" OR TI ("personality style*" OR "personality trait*" OR "attachment" OR "suggestibility" OR ("sensitivity" N3 "other*") OR "interoceptive awareness" OR "interoception*" OR "alexithymia" OR ("sense" N3 "agency") OR ("open*" N3 "experienc*") OR "agreeable*" OR "autonom* connect*") OR AB ("personality style*" OR "personality trait*" OR "attachment" OR "suggestibility" OR ("sensitivity" N3 "other*") OR "interoceptive awareness" OR "interoception*" OR "alexithymia" OR ("sense" N3 "agency") OR ("open*" N3 "experienc*") OR "agreeable*" OR "autonom* connect*") OR KW ("personality style*" OR "personality trait*" OR "attachment" OR "suggestibility" OR ("sensitivity" N3 "other*") OR "interoceptive awareness" OR "interoception*" OR "alexithymia" OR ("sens*" N3 "agenc*") OR ("open*" N3 "experienc*") OR ("harm*" N3 ("avoid*" OR "reduct*")) OR "agreeable*" OR "autonom* connect*")** | **165,261** |
| **S1** | **DE "Somatoform Disorders" OR DE "Conversion Disorder" OR DE "Factitious Disorders" OR DE "Dissociative Disorders" OR DE "Functional Paralysis" OR DE "Functional Visual Symptoms" OR DE "Pseudodementia" OR (DE "Movement Disorders" AND (TI ("functional") OR AB ("functional") OR KW ("functional"))) OR DE "Histrionic Personality Disorder" OR DE "Hysteria" OR DE "Catalepsy" OR TI ("conversion disorder*" OR "somatoform dis*" OR "functional tic*" OR "functional paralys*" OR "functional vis*" OR "hysterical paralys*" OR "hysterical vision disturbance*" OR "hysterical blindness" OR "hysteria*" OR "histrionic" OR "pseudodementia" OR "pseudo-dementia" OR "catalep*" OR "psychogenic nonepileptic seizur*" OR "psychogenic non-epileptic seizur*" OR "PNES" OR ("functional neurological" N3 "disorder*") OR "FND" OR "FNSD" OR "function* motor disorder*" OR "FMD") OR AB ("conversion disorder*" OR "somatoform dis*" OR "functional tic*" OR "functional paralys*" OR "functional vis*" OR "hysterical paralys*" OR "hysterical vision disturbance*" OR "hysterical blindness" OR "hysteria*" OR "histrionic" OR "pseudodementia" OR "pseudo-dementia" OR "catalep*" OR "psychogenic nonepileptic seizur*" OR "psychogenic non-epileptic seizur*" OR "PNES" OR ("functional neurological" N3 "disorder*") OR "FND" OR "FNSD" OR "function* motor disorder*" OR "FMD") OR KW ("conversion disorder*" OR "somatoform dis*" OR "functional tic*" OR "functional paralys*" OR "functional vis*" OR "hysterical paralys*" OR "hysterical vision disturbance*" OR "hysterical blindness" OR "hysteria*" OR "histrionic" OR "pseudodementia" OR "pseudo-dementia" OR "catalep*" OR "psychogenic nonepileptic seizur*" OR "psychogenic non-epileptic seizur*" OR "PNES" OR ("functional neurological" N3 "disorder*") OR "FND" OR "FNSD" OR "function* motor disorder*" OR "FMD")** | **30,380** |

**Supplementary References**

Cuoco, V., Gerace, C., Di Crosta, A., Mento, C., Cacciatore, M., Di Cara, G., Rifici, C., & Quattrini, F. (2021). *Attachment styles, identification of feelings, and psychiatric symptoms in functional neurological disorders.* *Journal of Psychosomatic Research, 147*, 110539. <https://doi.org/10.1016/j.jpsychores.2021.110539>

Demartini, B., Ricciardi, L., Crucianelli, L., Fotopoulou, A., & Edwards, M. J. (2016). *Sense of body ownership in patients affected by functional motor symptoms (conversion disorder).* *Consciousness and Cognition, 39*, 70–76. <https://doi.org/10.1016/j.concog.2015.11.005>

Ekanayake, V., Kranick, S., LaFaver, K., Naz, A., Webb, A. F., LaFrance, W. C., Jr., Hallett, M., & Voon, V. (2017). *Personality traits in psychogenic nonepileptic seizures (PNES) and psychogenic movement disorder (PMD): Neuroticism and perfectionism.* *Journal of Psychosomatic Research, 97*, 23–29. <https://doi.org/10.1016/j.jpsychores.2017.03.018>

Erten, E., Yenilmez, Y., Fistikci, N., & Saatcioglu, O. (2013). *The relationship between temperament and character in conversion disorder and comorbid depression.* *Comprehensive Psychiatry, 54*(4), 354–361. <https://doi.org/10.1016/j.comppsych.2012.10.007>

Gerhardt, A., Schramm, A., van der Ven, A., Doering, B. K., Kyndt, C., Vlerick, P., & Mertens, V. C. (2021). *Insecure and unresolved/disorganized attachment in patients with psychogenic nonepileptic seizures.* *Journal of the Academy of Consultation-Liaison Psychiatry, 62*(3), 337–344. <https://doi.org/10.1016/j.psym.2020.05.014>

Huys, A.-C. M. L., Beck, B., Haggard, P., Bhatia, K. P., & Edwards, M. J. (2021). *No increased suggestibility to placebo in functional neurological disorder.* *European Journal of Neurology, 28*(7), 2367–2371. <https://doi.org/10.1111/ene.14816>

Huys, A.-C. M. L., Edwards, M. J., Bhatia, K. P., & Haggard, P. (2020). *Modulation of reaction times and sense of agency via subliminal priming in functional movement disorders.* *Frontiers in Neurology, 11*, 989. <https://doi.org/10.3389/fneur.2020.00989>

Jalilianhasanpour, R., Ospina, J. P., Williams, B., Mello, J., MacLean, J., Ranford, J., Fricchione, G. L., LaFrance, W. C., Jr., & Perez, D. L. (2019). *Secure attachment and depression predict 6-month outcome in motor functional neurological disorders: A prospective pilot study.* *Psychosomatics, 60*(4), 365–375. <https://doi.org/10.1016/j.psym.2018.08.004>

Koreki, A., Garfinkel, S. N., Mula, M., Agrawal, N., Cope, S., Eilon, T., Duncan, R., Reuber, M., & Critchley, H. D. (2020). *Trait and state interoceptive abnormalities are associated with dissociation and seizure frequency in patients with functional seizures.* *Epilepsia, 61*(6), 1156–1165. <https://doi.org/10.1111/epi.16532>

Krámská, L., Hrešková, L., Krámský, D., & Vojtěch, Z. (2022). *Attachment style of patients diagnosed with psychogenic non-epileptic seizures at a tertiary epilepsy center in the Czech Republic.* *Frontiers in Psychiatry, 13*, 1065201. <https://doi.org/10.3389/fpsyt.2022.1065201>

Kranick, S. M., Moore, J. W., Yusuf, N., Martinez, V. T., LaFaver, K., Edwards, M. J., Weir, J. P., & Hallett, M. (2013). *Action-effect binding is decreased in motor conversion disorder: Implications for sense of agency.* *Movement Disorders, 28*(8), 1110–1116. <https://doi.org/10.1002/mds.25408>

Leong, M., Wang, A. D., Trainor, D., Johnstone, B., Rayner, G., Kalincik, T., Roos, I., Kwan, P., O'Brien, T. J., Velakoulis, D., & Malpas, C. B. (2019). *Personality profiles differ between patients with epileptic seizures and patients with psychogenic non-epileptic seizures.* *Seizure, 73*, 1–8. <https://doi.org/10.1016/j.seizure.2019.10.011>

Levita, L., Mayberry, E., Mehmood, A., & Reuber, M. (2020). *Evaluation of LiNES: A new measure of trauma, negative affect, and relationship insecurity over the life span in persons with FND.* *The Journal of Neuropsychiatry and Clinical Neurosciences, 32*(1), 43–49. <https://doi.org/10.1176/appi.neuropsych.19050121>

Macerollo, A., Chen, J.-C., Pareés, I., Kassavetis, P., Kilner, J. M., & Edwards, M. J. (2015). *Sensory attenuation assessed by sensory evoked potentials in functional movement disorders.* *PLOS ONE, 10*(6), e0129507. <https://doi.org/10.1371/journal.pone.0129507>

Marotta, A., Bombieri, F., Zampini, M., Schena, F., Dallocchio, C., Fiorio, M., & Tinazzi, M. (2017). *The moving rubber hand illusion reveals that explicit sense of agency for tapping movements is preserved in functional movement disorders.* *Frontiers in Human Neuroscience, 11*, 291. <https://doi.org/10.3389/fnhum.2017.00291>

Millman, L. S. M., Short, E., Stanton, B., Winston, J. S., Nicholson, T. R., Mehta, M. A., Kanaan, R. A. A., & David, A. S. (2023). *Interoception in functional motor symptoms and functional seizures: Preliminary evidence of intact accuracy alongside reduced insight and altered sensibility.* *Behaviour Research and Therapy, 168*, 104379. <https://doi.org/10.1016/j.brat.2023.104379>

Morris, L. S., To, B., Baek, K., Chang-Webb, Y.-C., Mitchell, S., Strelchuk, D., Irvine, M. A., Robbins, T. W., Wood, J., Harrison, N. A., & Voon, V. (2017). *Disrupted avoidance learning in functional neurological disorder: Implications for harm avoidance theories.* *NeuroImage: Clinical, 16*, 286–294. <https://doi.org/10.1016/j.nicl.2017.08.007>

Nahab, F. B., Kundu, P., Maurer, C., Shen, Q., & Hallett, M. (2017). *Impaired sense of agency in functional movement disorders: An fMRI study.* *PLoS ONE, 12*(4), e0172502. <https://doi.org/10.1371/journal.pone.0172502>

Pareés, I., Brown, H., Nuruki, A., Adams, R. A., Davare, M., Bhatia, K. P., Friston, K. J., & Edwards, M. J. (2014). *Loss of sensory attenuation in patients with functional (psychogenic) movement disorders.* *Brain, 137*(11), 2916–2921. <https://doi.org/10.1093/brain/awu237>

Pick, S., Rojas-Aguiluz, M., Butler, M., Mulrenan, H., Nicholson, T. R., & Goldstein, L. H. (2020). *Dissociation and interoception in functional neurological disorder.* *Cognitive Neuropsychiatry, 25*(4), 294–311. <https://doi.org/10.1080/13546805.2020.1791061>

Ricciardi, L., Demartini, B., Crucianelli, L., Krahe, C., Edwards, M. J., & Fotopoulou, A. (2016). *Interoceptive awareness in patients with functional neurological symptoms.* *Biological Psychology, 113*, 68–74. <https://doi.org/10.1016/j.biopsycho.2015.10.009>

Roelofs, K., Keijsers, G. P. J., Hoogduin, K. A. L., Näring, G. W. B., & Moene, F. C. (2002). *Hypnotic susceptibility in patients with conversion disorder.* *Journal of Abnormal Psychology, 111*(2), 390–395. <https://doi.org/10.1037/0021-843X.111.2.390>

Sarisoy, G., Kaçar, Ö. F., Öztürk, A., Yilman, T., Mor, S., Özturan, D. D., Yazici, N., & Gümüş, K. (2015). *Temperament and character traits in patients with conversion disorder and their relations with dissociation.* *Psychiatria Danubina, 27*(4), 390–396.

Sojka, P., Diez, I., Bareš, M., & Perez, D. L. (2021). *Individual differences in interoceptive accuracy and prediction error in motor functional neurological disorders: A DTI study.* *Human Brain Mapping, 42*(5), 1434–1445. <https://doi.org/10.1002/hbm.25304>

Tinazzi, M., Marotta, A., Zenorini, M., Riello, M., Antonini, A., & Fiorio, M. (2021). *Movement perception of the tonic vibration reflex is abnormal in functional limb weakness.* *Parkinsonism & Related Disorders, 87*, 1–6. <https://doi.org/10.1016/j.parkreldis.2021.04.004>

Willinger, U., & Aschauer, H. N. (2005). *Personality, anxiety and functional dysphonia.* *Personality and Individual Differences, 39*(8), 1441–1449. <https://doi.org/10.1016/j.paid.2005.06.011>

Williams, B., Ospina, J. P., Jalilianhasanpour, R., Fricchione, G. L., & Perez, D. L. (2019). *Fearful attachment linked to childhood abuse, alexithymia, and depression in motor functional neurological disorders.* *The Journal of Neuropsychiatry and Clinical Neurosciences, 31*(1), 65–69. <https://doi.org/10.1176/appi.neuropsych.18040095>

Williams, I. A., Reuber, M., & Levita, L. (2021). *Interoception and stress in patients with functional neurological symptom disorder.* *Cognitive Neuropsychiatry, 26*(2), 75–94. <https://doi.org/10.1080/13546805.2020.1865895>
